# Problem Solved? An Individual Ratio between Point-of-Care and Venous International Normalized Ratio Values in Two Patients with Antiphospholipid Syndrome: Two Case Reports

**DOI:** 10.1055/a-2542-5358

**Published:** 2025-03-19

**Authors:** Bettina C. Geertsema-Hoeve, Massimo Radin, Savino Sciascia, Rolf T. Urbanus, Albert Huisman, Josine Borgsteede-de Wilde, Maarten Limper

**Affiliations:** 1 Division Internal Medicine and Dermatology, Department of Clinical Immunology and Rheumatology, University Medical Center Utrecht, Utrecht, The Netherlands; 2 Department of Clinical and Biological Sciences, University of Torino, Torino, Italy; 3 Division Laboratories, Pharmacy and Medical Sciences, Central Diagnostic Laboratory, University Medical Center Utrecht, Utrecht, The Netherlands

**Keywords:** antiphospholipid syndrome, International Normalized Ratio, point-of-care testing, vitamin-k antagonists, case report

## Abstract

Antiphospholipid syndrome (APS) is a rare autoimmune disorder characterized by thromboembolic and obstetric complications in the presence of persistent antiphospholipid antibodies (aPL). Treatment aims to prevent recurrent thrombosis, primarily using anticoagulation therapy with vitamin K antagonists (VKA). Monitoring of VKA therapy relies on the International Normalized Ratio (INR), which can be assessed using point-of-care testing (POCT). However, in a subset of APS patients with a high-risk aPL profile, the POCT-INR is falsely elevated, which might lead to underdosing of VKA and subsequent high risk of recurrent thrombosis. This case report describes two female patients with triple-positive thrombotic APS receiving VKA therapy. Both patients underwent biweekly paired INR measurements via POCT-INR and venous INR methods. Despite significant discrepancies, a strong individual linear correlation was observed: *r* = 0.77 (95% confidence interval [CI]: 0.54–0.99, *p* < 0.001) and *r* = 0.93 (95% CI: 0.88–0.97, *p* < 0.001), respectively. These findings suggest that individualized correction factors could be developed to improve the accuracy of POCT-INR measurements, thereby optimizing VKA dosing in these patients.

## Introduction

Antiphospholipid syndrome (APS) is a rare autoimmune disorder characterized by the persistent presence of antiphospholipid antibodies (aPL) and recurrent thrombotic events, both macro- and microvascular, as well as pregnancy-related complications. The key aPL subtypes implicated in APS are anticardiolipin antibodies, anti-β2-glycoprotein I antibodies, and lupus anticoagulant (LA). 1 The increased thrombotic risk in APS is attributed to dysregulation of the coagulation cascade and endothelial dysfunction. 2 Treatment primarily involves anticoagulation with vitamin K antagonists (VKA), such as warfarin or phenprocoumon to prevent recurrent thrombotic events. 3 The efficacy of VKA therapy is monitored using the International Normalized Ratio (INR), which reflects the ratio of a patient's prothrombin time to a standardized prothrombin time. 4 The typical therapeutic INR range is 2.0 to 3.0, with higher targets (e.g., 2.5–3.5) recommended for patients with recurrent thrombotic events. 3 Maintaining the INR within this narrow therapeutic range is critical, as values outside this range increase the risk of either thrombosis or bleeding. 4 Point-of-care testing (POCT)-INR provides rapid and convenient INR monitoring, especially in outpatient or home-based settings. 5 However, in APS patients, POCT-INR results are often less accurate than laboratory-based venous INR measurements. This inaccuracy is attributed to the interference of aPL with phospholipid-dependent coagulation assays, which may lead to overestimation of POCT-INR values. 6 7 Such discrepancies can result in suboptimal anticoagulation management, exposing patients to the risk of thrombotic or bleeding. Given these limitations, strategies to enhance INR monitoring accuracy in APS patients are essential. One promising approach is the development of individualized correction factors to adjust POCT-INR values, thereby improving their reliability for VKA dose management in this high-risk population.

## Methods

In two patients with a known discrepancy between venous- and POCT-INR, biweekly paired INR measurements by means of serial point-of-care (POC)-INR and venous INR measurements were registered. Blood samples for POCT-INR (CoaguChek XS) were obtained from a finger stick puncture in accordance with the CoaguChek XS (Roche Diagnostics, Rotkreuz, Switzerland) manufacturer's guidelines. Each blood sample was analyzed with the CoaguChek XS device immediately after the finger stick. Within 1 hour after the finger stick puncture, phlebotomy was performed to draw citrate blood samples (3.2% sodium citrate blood collection tubes, Vacuette, Greiner BioOne, Kremsmünster, Austria) for determining the INR. Plasma was obtained by centrifugation at 4000 × g for 5 minutes. The INR of the first patient was measured with HemosIL products (Werfen, RecombiPlasTin 2G and ReadiPlasTin) for PT, and that of the second patient on an ACL top 750 LAS coagulation analyzer (Werfen, Barcelona, Spain) with a prothrombin time based on Owren's method with rabbit brain–derived thromboplastin (Technoclot PT Owren, Technoclone, Vienna, Austria). Correlation was assessed by means of the Pearson's correlation test and an individual linear *y* -formula was constructed per patient.

## Results

One patient from Italy and one from the Netherlands, both with triple-positive APS, were included. The first patient, a 56-year-old female diagnosed at the age of 31, had a history of pregnancy complications, multiple deep venous thromboses (some during VKA therapy), a lacunar stroke, and livedo reticularis. She home-monitored her INR with a CoaguChek XS POC-INR device. Despite anticoagulation with low-dose aspirin and warfarin with a target INR higher than normal (3–4), she experienced ongoing thrombotic events. The second patient, a 48-year-old female diagnosed at the age of 39, had a history of recurrent pulmonary embolism and deep venous thrombosis despite anticoagulation with phenprocoumon. She also exhibited livedo reticularis. Due to persistent thrombotic events, an elevated target INR range of 3 to 4 was established. She used POCT with a CoaguChek device. A notable discrepancy was found when her POCT-INR was 6.8 while her venous INR, measured with the Owren method, was 3.0. Consequently, venous INR was used for dosing adjustments, although she continued to track POCT-INR for personal interest. The discrepancy between both measurements was clearly evident, particularly at higher INR levels.

**Fig. 1:**
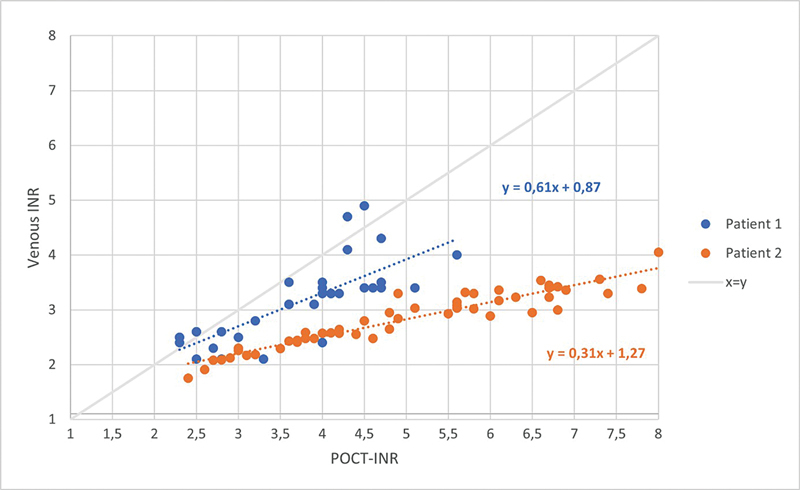
Correlation between POCT-INR and venous INR values in two patients with antiphospholipid syndrome. The results of serial paired measurements of POCT-INR and venous INR, measured by Owren's method. Although the underlying mathematical formulas differ, both patients exhibited a linear correlation between POCT-INR and venous INR. Y formula is the formula with which a venous INR can be calculated from a POCT-INR value. Pearson's correlation for patient 1 was *r* = 0.77 ( *p* < 0.001), Pearson's correlation for patient 2 was *r* = 0.93 ( *p* < 0.001). INR, International Normalized Ratio; POCT, point-of-care testing.

Biweekly paired INR measurements were obtained for 17 (patient 1) and 26 (patient 2) weeks ( Fig. 1 ). POCT-INR values correlated well with venous INR values for both patient 1 ( *r* = 0.77 [95% confidence interval: 0.54–0.99, bootstrap, *p* < 0.001]) and patient 2 ( *r* = 0.93 [95% confidence interval 0.88–0.97, bootstrap, *p* < 0.001]), suggesting personalized correction of the POCT-INR to reflect the venous INR is feasible.

## Discussion

The observed discrepancy between POCT-INR and venous INR measurements in our cases underscores the challenges with accurate INR monitoring for APS patients. Our findings, especially at higher INR levels, align with previous reports suggesting that aPL can interfere with phospholipid-dependent coagulation reactions, particularly in POCT platforms, leading to overestimation of INR values. 6 This interference, noted particularly in LA-positive APS patients with high anti-β2-glycoprotein I titers, 6 raises concerns about the reliability of POCT-INR for guiding VKA therapy in these patients, potentially increasing the risk of thromboembolic events. However, given that estimated 33% of APS patients are triple positive, 8 this interference is relevant for a significant proportion of patients. Previous research indicated that the likelihood of discrepancy is higher in triple-positive patients; 6 however, it has not yet been fully clarified for which subset of patients this applies and what distinguishes these patients from triple-positive individuals who do not experience discrepancies.

A potential cause for the discrepancy between POCT-INR and venous INR lies in the used reagents. In the POC assay, human recombinant thromboplastin is used, which appears to be more sensitive to variations in the presence of LA. 9 Moreover, the POCT-INR does not measure clot formation but instead assesses thrombin generation using an electrically conductive substrate. 10 For determining the INR in patients treated with VKAs, the INR Owren method appears to have superior clinical performance with lower interlaboratory variability compared with the INR Quick method. 11 12 13 In addition to the reagent, the dilution factor may also have an impact on the INR results. The dilution factor used in the venous measurement method, the Owren method, is comparatively high, making this assay less susceptible to (LA) interference. 14 Following the identification of this discrepancy, it was decided within the respective subset of APS patients to revert solely to venous measurements to ensure the safety of VKA administration. However, this approach is time-consuming, more painful, and diminishes the quality of life of these patients. Demonstrating the ability to calculate a safe correction factor per patient and thereby reestablishing access to POCT-INR measurements would constitute a significant advancement in enhancing the quality of life for these patients with a chronic condition.

The key finding in these cases is that POCT consistently measures higher INR levels compared with venous testing, with a predictable positive linear correlation, suggesting the potential to develop individualized correction factors to adjust POCT-INR values for more accurate VKA dosing. By accounting for the inherent variability in POCT-INR measurements introduced by aPL interference, clinicians could potentially optimize VKA therapy while minimizing thromboembolic risks in APS patients. However, the development and validation of such correction factors necessitate further investigation in larger patient cohorts, considering the heterogeneity of APS presentations. The relatively wide 95% confidence interval for the *r* -value of one of the patients might be attributed to the impact of aPL titers, which likely influenced the measurements and should be accounted for in future research, including the assessment of specific aPL subtypes, their titers, and potential interactions with coagulation factors. Since it has previously been shown that aPL can induce LA by activation of coagulation factors, 15 these interactions should be researched more specifically to determine the extent of these interactions on the actual INR results. Additionally, the inherent differences between the testing methods may contribute to this variability, further underscoring the need for careful calibration and investigation in subsequent studies.

## Conclusion

Our cases highlight the challenges associated with INR monitoring in APS patients undergoing VKA therapy, particularly regarding the reliability of POCT-INR measurements. POCT-INR values can overestimate INR compared with venous measurements, due to the presence of aPL in the triple-positive APS patient. Therefore, we recommend measuring INR using both POCT and venous (Owren method) at the initial assessment to compare results. If a significant discrepancy is observed, it may be advisable to rely solely on venous INR measurement for further monitoring. The observed linear correlation between these two measurements suggests the feasibility of developing individualized correction factors to improve the accuracy of POCT-INR for VKA dosing in these patients. However, further research in more patients is warranted to validate these correction factors and to clarify the underlying mechanisms of aPL interference with coagulation assays in APS patients. An ongoing study in Utrecht, the Netherlands, is currently exploring this issue in a larger cohort of APS patients. Ultimately, optimizing INR monitoring strategies is of great importance to ensure safe and effective anticoagulation therapy in APS patients, thereby minimizing thromboembolic and bleeding risks while enhancing clinical outcomes.
